# A Porous Carbon with Excellent Gas Storage Properties from Waste Polystyrene

**DOI:** 10.3390/nano9050726

**Published:** 2019-05-10

**Authors:** Giorgio Gatti, Mina Errahali, Lorenzo Tei, Enzo Mangano, Stefano Brandani, Maurizio Cossi, Leonardo Marchese

**Affiliations:** 1 Dipartimento di Scienze e Innovazione Tecnologica (DISIT), Università del Piemonte Orientale, via T. Michel 11, I-15121 Alessandria, Italy; mina.errahali@uniupo.it (M.E.); lorenzo.tei@uniupo.it (L.T.); maurizio.cossi@uniupo.it (M.C.); leonardo.marchese@uniupo.it (L.M.); 2 School of Engineering, Universtity of Edinburgh, Sanderson Building, R. Stevenson Road, Edinburgh EH11 3AZ, UK; e.mangano@ed.ac.uk (E.M.); s.brandani@ed.ac.uk (S.B.)

**Keywords:** porous carbon, hyper-crosslinked polymer, methane storage, waste valorization

## Abstract

In this paper, we describe the synthesis and gas adsorption properties of a porous carbonaceous material, obtained from commercial expanded polystyrene. The first step consists of the Friedel-Craft reaction of the dissolved polystyrene chains with a bridging agent to form a highly-crosslinked polymer, with permanent porosity of 0.7 cm^3^/g; then, this polymer is treated with potassium hydroxide at a high temperature to produce a carbon material with a porous volume larger than 1.4 cm^3^/g and a distribution of ultramicro-, micro-, and mesopores. After characterization of the porous carbon and determination of the bulk density, the methane uptake was measured using a volumetric apparatus to pressures up to 30 bar. The equilibrium adsorption isotherm obtained is among the highest ever reported for this kind of material. The interest of this product lies both in its excellent performance and in the virtually costless starting material.

## 1. Introduction

The numerous applications of methane as an energy vector would benefit greatly from adsorbed natural gas (ANG) technologies [1,2,3], which are expected to compensate for the relatively low energy density of gaseous methane reducing the costs related to compressed or liquified natural gas (CNG or LNG, respectively) approaches. With suitable adsorbers, the gas storage pressure in tanks could be lowered up to 20% of the value necessary in CNG [4], or alternatively, the stored quantity could be largely increased at the same pressure. These expectations have been quantified by the U.S. Department of Energy (DOE) with the storage target of 180 cm^3^(STP)/cm^3^ at 35 bar (where STP stands for standard temperature and pressure, 273 K and 1 bar), raised successively to 263 cm^3^(STP)/cm^3^, though it is a value thought to be too ambitious by some researchers [5].

Among the various classes of materials proposed for ANG applications [6,7,8,9,10,11,12,13,14,15], activated porous carbons seem the most promising for several reasons [3,4,16,17,18,19,20,21]: versatility, which allows for tuning of the porosity by tailoring the starting materials and the reaction conditions; high mechanical and chemical stability; and finally, the possibility to design cheap and scalable productions.

Here, we describe the preparation and characterization of a porous carbon with a very high methane uptake capacity, obtained through an easy and scalable reaction from expanded polystyrene (a virtually costless material which can be recycled from the packaging industry). The very good performances of this adsorber are related to the favorable pore distribution in the regions of micropores (7–20 Å) and small mesopores (20–50 Å) coupled with high bulk density, leading to an unusually large uptake on a volume basis.

The material was prepared in two steps: first, a hyper-crosslinked polymer (HCP) was obtained with the Friedel-Crafts reaction from expanded polystyrene (PS): this material is referred to as HCP-PS in the following. HCP have been widely studied [22,23,24,25,26,27,28] for their simplicity of synthesis [29,30,31] and excellent mechanical and textural properties. Cooper et al. studied the crosslinking reaction of various polystyrenes synthesized in situ with different molecular weights; Fu et al. [32] reported the same reaction on expanded PS, using 1,2-dichloroethane as the crosslinking agent [33]. Very recently, waste polystyrene foam was used to prepare cross-linked polymers through a Friedel-Crafts reaction with acetone and aluminum chloride in the presence of silica nanoparticles, followed by calcination to obtain a porous carbon [34]; another porous carbon was prepared from PS by pyrolysis and chemical activation with KOH [35]. Then, HCP-PS was chemically and thermally activated, producing a highly porous carbonaceous material, named KPS-1. Analogous procedures were recently employed to prepare porous carbons from HCP [36,37,38,39,40]. We have also followed this approach in the past starting from tetraphenylmethane, testing different reaction conditions to optimize the textural properties of the products [31,41,42].

## 2. Materials and Methods

**Figure 1:**
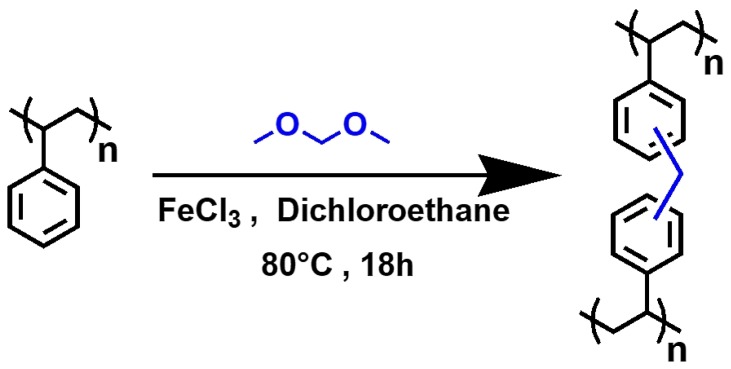
Formation of hyper-crosslinked polymeric material from polystyrene with the Friedel-Crafts reaction using formaldehyde dimethyl acetal (FDA) as a linker.

As mentioned above [31,32] and illustrated in Figure 1, the hyper-crosslinked polymer HCP-PS was obtained using the Friedel-Crafts reaction from expanded polystyrene (PS), using ferric chloride as a catalyst and formaldehyde dimethyl acetal (FDA) as a bridging agent. 20 g of polystyrene from packaging (PS) and 93.1 g of FeCl_3_ (0.57 mol) were suspended in 0.54 L of dichloroethane (DCE). The mixture was stirred mechanically at room temperature to obtain a homogeneous solution; then, 54 mL (0.61 mol) of formaldehyde dimethylacetal (FDA) were added dropwise. The mixture was stirred at room temperature for 30 min and then heated at 80 °C for 18 h. After cooling to room temperature, the thick gel was diluted with ethanol and washed several times with EtOH and water until a neutral pH was reached. Finally, the solid was dried in the oven at 110 °C overnight (the material obtained is named HCP-PS).

To obtain the porous carbon, 2 g of HCP-PS were homogeneously grinded with 6 g of KOH (0.107 mol) under inert conditions. Then, the mixture was placed in a crucible of alumina and thermally treated under a N_2_ flow with a ramp of 2 °C/min up to 800 °C, and subsequently held under isothermal conditions for 1 h. After the chemical activation, [23] all the resulting carbons were washed with deionized water (250 mL), with 2M HCl (200 mL) and washed again with deionized water to remove potassium salts and to neutralize the solution, and then dried at 110 °C for 18 h (the final material was named KPS-1).

The materials were characterized by Fourier Transform Infrared (FTIR) spectra, recorded with a Brucker Equinox 55 spectrometer (Billerica, USA), equipped with a Deuterated triglycine sulfate detector (DTGS) pyroelectric detector with 4 cm^−1^ resolution, and Raman spectra, recorded as prepared powders using a Jobin Yvon HR800 Lab-Ram μ-spectrometer (Kyoto, Japan), equipped with an Olympus BX41 microscope, a He-Ne 20 mW laser working at 632.8 nm, and a charge-coupled device (CCD).

The pore structures of HCP-PS and KPS-1 were investigated by adsorbing N_2_ at 77 K and CO_2_ at 273 K and analyzing the adsorption isotherms using the Quenched Solid Density Functional Theory (QSDFT) method (using carbon slit/cylindrical pore parameters) [43,44].

Solid-state Nuclear Magnetic Spectra (NMR) spectra were acquired on a Bruker Avance III 500 spectrometer (Billerica, USA), and a wide bore 11.7 Tesla magnet with operational frequencies for ^1^H and ^13^C of 500.13 and 125.77 MHz, respectively. A 2.5 mm double resonance probe with Magic Angle Spinning (MAS) was employed in all experiments. The magnitude of radio frequency field was 131 kHz for ^1^H MAS NMR and relaxation delays, d1, between accumulations was 3 s. For the ^13^C cross-polarization (CP) magic angle spinning (MAS) experiments, the proton radio frequency (RF) of 100 and 42 kHz were used for initial excitation and decoupling, respectively.

During the CP period, the ^1^H RF field was ramped using 100 increments, whereas the ^13^C RF field was maintained at a constant level. During the acquisition, the protons were decoupled from the carbons by using a Spinal64 decoupling scheme. A moderate ramped RF field of 54 kHz was used for spin-locking, while the carbon RF field was matched to obtain an optimal signal and CP contact times of 0.5 ms were used. The ^13^C NMR spectra were also acquired using DP (direct polarization) MAS-based experiments under high-power proton-decoupling conditions.

To avoid baseline distortions and to remove ^13^C background signals associated with the probe in direct polarization magic angle spinning (DPMAS) experiments, a special pulse program (aringdec) was used from the Bruker topspin library. The magnitude of the radio frequency field was 80 kHz for ^13^C MAS NMR, and the ^1^H decoupling field was 66 kHz, as well as the relaxation delays, d1, between accumulations was 30 s. All chemical shifts were reported using the δ scale and externally referenced to tetramethylsilane at 0 ppm.

## 3. Results and Discussion

### 3.1. Vibrational Characterization

**Figure 2:**
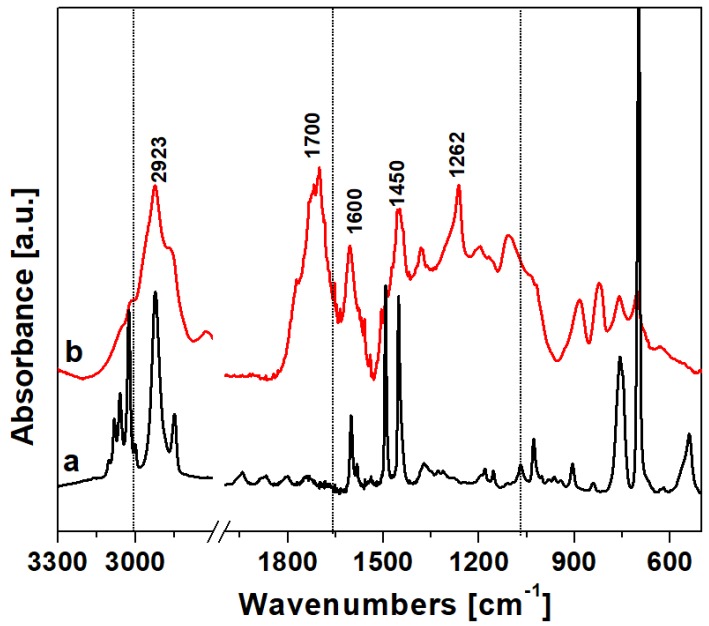
Vibrational spectra in the region 3300–500 cm^−1^ of (a) the hyper-crosslinked polymer polymer and (b) polystyrene precursor.

The FTIR spectrum of HCP-PS is reported in Figure 2. In the high-frequency region, both the PS precursor (curve a) and the HCP (curve b) are characterized by bands at 3100–3000 cm^−1^, attributed to aromatic C–H groups stretching modes. After polymerization, the group of bands in the region 3000–2800 cm^−1^ due to the stretching of aliphatic C–H became more intense due to methylene linkers between the aromatic rings. The low-frequency region shown in the PS spectrum the structural bands were due to –CH_2_ and aromatic –CH_2_ bending bands. The spectrum of HCP shows new bands in the region 1800–1700 cm^−1^ and 950–650 cm^−1^ due to the higher number of positions substituted on the aromatic rings after the polymerization. Also, the bands at 1450 cm^−1^ due to the –CH_2_ bending mode of the methylene linker, along with the band 1262 cm^−1^ due to –CH_2_ wagging modes of the chloro-methylene groups confirm the successful cross-linking reaction.

**Figure 3:**
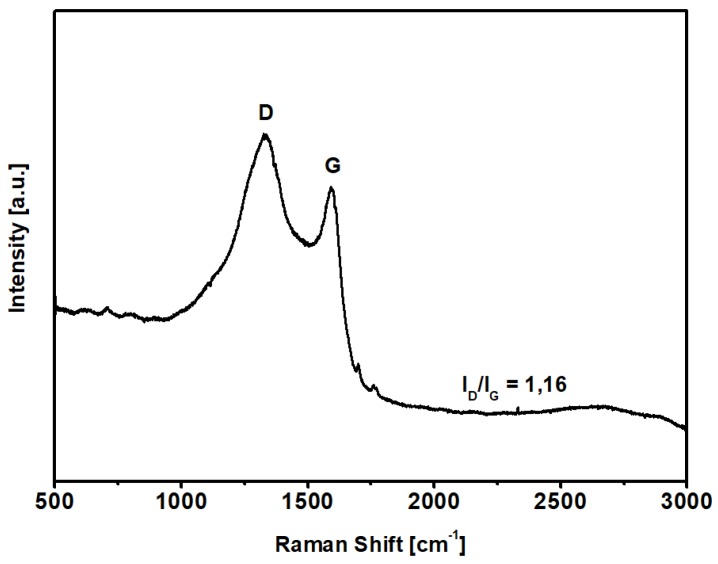
Raman spectrum of the carbon sample in the region 3000–500 cm^−1^.

Raman spectroscopy was used to evaluate the carbonization degree after the KOH-activated thermal process described above; the spectrum of KPS-1 is shown in Figure 3. The KPS-1 Raman spectrum shows two main vibrational modes at 1329 and 1593 cm^−1^. The 1593 cm^−1^ mode (G peak) corresponds to the Raman-allowed $E_{2g}$ mode in the ideal graphite, and the 1329 cm^−1^ mode (D peak) corresponds to the disorder-induced band, which is associated to the large density of phonon states [45]. In particular, the strong D-band peak demonstrates that the microporous carbon has a low degree of graphitization ($I_{D}/I_{G}$ = 1.16) and contains a significant amount of disordered sections and defects [46].

### 3.2. Pore-Size Analysis

To evaluate the textural properties of the synthesized materials, we measured the adsorption isotherms of nitrogen at 77 K and carbon dioxide at 293 K, both in HCP-PS and in KPS-1. With N_2_, HCP-PS shows a type IV isotherm (with specific surface area, SSA_BET_, of 739 m^2^/g) with a pronounced hysteresis loop between 0.9 and about 0.4 P/P_0_, evidencing the presence of mesopores in the material. On the other hand, the derivated carbon KPS-1 shows a pseudo-Langmuir isotherm of type I (SSA_BET_ = 2637 m^2^/g) and a small hysteresis loop of type H4 that can be associated to the presence of narrow slit pores, as commonly found for nitrogen sorption isotherms on activated carbons. The porosity of both the materials was described in greater detail by applying QSDFT analysis to the adsorption isotherms.

**Figure 4:**
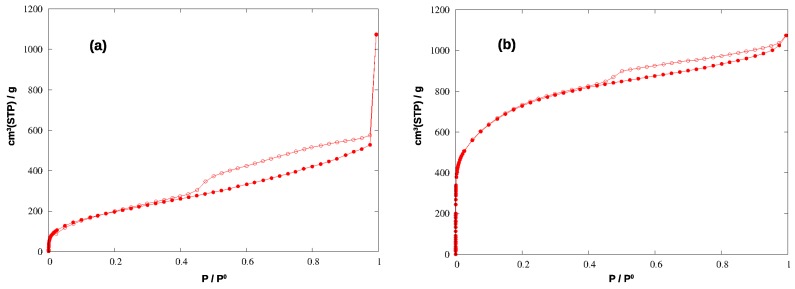
Adsorption (filled circles) and desorption (empty circles) isotherms of N_2_ at 77 K in the hyper-crosslinked polymer (**a**) and in the carbonized material (**b**).

**Figure 5:**
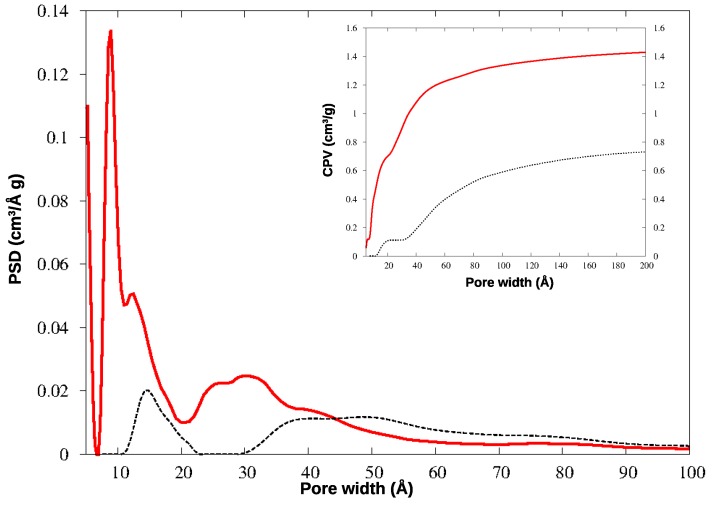
Pore size distribution (PSD) and cumulative pore volume (CPV, insert) for HCP-PS (black dotted line) and KPS-1 (red solid line) from N_2_ adsorption at 77 K.

The N_2_ adsorption isotherms for the two materials are shown in Figure 4, and the corresponding pore size distributions (PSD) and cumulative pore volumes (CPV) are compared in Figure 5.

**Figure 6:**
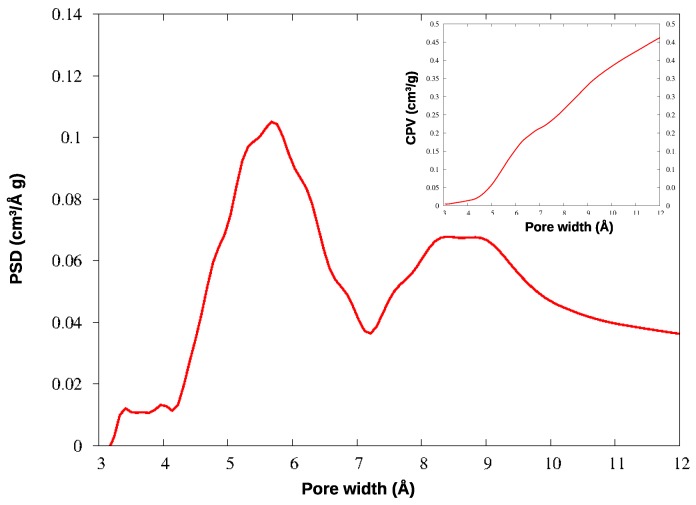
PSD and CPV for KPS-1 from CO_2_ adsorption at 273 K.

In KPS-1, a rich family of micropores appears below 10 Å. To investigate this region in greater detail, this material was further analyzed by adsorbing CO_2_ at 273 K: the QSDFT analysis was applied to the CO_2_ isotherm, providing the PSD and CPV for ultramicropores, reported in Figure 6. The smallest pores have been described in much greater detail by CO_2_ adsorption at the higher temperature, as is well-known: the ultramicropores around 4–7 Å width are clearly characterized by this experiment. Notably, both gases find a family of micropores at 9 Å, though their relative abundance is different in the N_2_ and CO_2_ curves; however, the two gases provide very similar values for the total microporous volume (CPV).

HCP-PS presents a porous volume of 0.74 cm^3^/g, of which 0.11 cm^3^/g is due to micropores (below 20 Å width): the analogous systems prepared by the Friedel-Crafts reaction from tetraphenylmethane, in different conditions, showed porous volumes in the range 0.64–0.83 cm^3^/g, with microporous contributions from 0.16 to 0.21 cm^3^/g [31]. Other HCP obtained from benzene, thiophene, and pyrrole were found to have porous volumes of 1.52 (0.40), 0.33 (0.22), and 0.25 (0.14) cm^3^/g, respectively (in parentheses are the microporous volumes) [16].

After the chemical and thermal activation, the porosity increased significantly, exceeding 1.40 cm^3^/g (note that pores above 200 Å width, approximately, become less and less important in gas adsorption processes), and the pore distribution changes deeply too. The same effect was reported after the carbonization of the HCP polymers mentioned above, where their porous volume increased to 1.58 (for benzene monomer), 1.51 (thiophene), and 3.14 (pyrrole) cm^3^/g [16].

The micropores in HCP-PS appear in a quite narrow distribution around 15 Å, while the mesopore family is widely spread above 30 Å width. In KPS-1, on the other hand, micropores split in two narrow peaks around 9 and 13 Å, and ultramicropores appear also, between 5 and 6 Å, as clearly shown by CO_2_ PSD in Figure 6. Overall, the KPS-1 microporous volume reaches 0.7 cm^3^/g; a large family of mesopores appears between 20 and 40 Å, adding 0.4 cm^3^/g to the porous volume, while a broad distribution of mesopores exist with a larger size, contributing approximately 0.32 cm^3^/g to the total volume. The porous volumes of the materials are resumed in Table 1, and compared to the volumes recently reported for other porous carbons obtained from PS.

### 3.3. Solid State NMR

**Figure 7:**
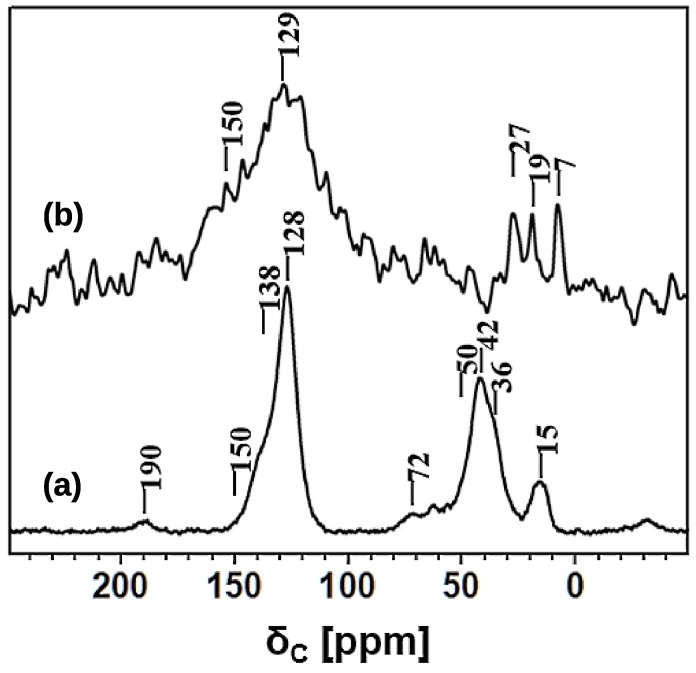
^13^C NMR spectra: (a) HCP-PS recorded using the CPMAS technique with a CP contact time of 0.5 ms; (b) KPS-1 recorded using the DPMAS technique with a MAS rate of 30 kHz.

The nature of hyper-crosslinking in HCP-PS was also investigated using solid-state ^13^C and ^1^H NMR spectroscopy. The HCP-PS ^13^C CPMAS NMR spectrum, recorded using 0.5 ms cross polarization contact time and 20 kHz MAS rate, is shown in Figure 7.

Aromatic ^13^C peaks in the range of 120–150 ppm were assigned as following: 128 ppm for the aromatic C–H and 138 ppm for the methylene- and/or methine-substituted aromatic carbons [31,47]; there are no ^13^C resonances above 145 ppm, confirming the absence of free polystyrene units in HCP-PS [48].

In the aliphatic region, the ^13^C resonances at 42 and 36 ppm were assigned to the methylene units of the polystyrene blocks and to the CH_2_ (benzylic carbon) linker. Polystyrene methine carbons appeared as a very broad resonance centered at 50 ppm, and this assignment was further supported by the intensity increase in the long contact-time CPMAS experiment (data not shown). Additional resonance at 72 ppm, is assigned to CH_2_OH groups, substituted on the aromatic rings [31]. On the other hand, CH_2_Cl carbons formed in the benzene chloromethylation during Friedel-Crafts reaction were expected at 43 ppm, and their presence cannot be ruled out. Moreover, a strong broad peak was also observed at 15 ppm and assigned to a CH_3_ group due to the methylation and/or ethylation of aromatic rings [49]; the unusual broad peak observed at around 190 ppm is tentatively assigned to carbonyl carbons.

Likewise, the ^13^C NMR spectrum was recorded on carbonized material, KPS-1. In this case, we employed the ^13^C DPMAS NMR technique, since the low proton content and the influence of paramagnetic interactions in KPS-1 are problematic for recording a ^13^C NMR spectrum using the CPMAS technique [50].

Indeed, a combination of several technical challenges, such as the highly electrically conductive nature of carbonized material, the magnetic susceptibility effects observed in graphitic material, the very low isotopic abundance of ^13^C, very long carbon recycle delay, and the low sample volume (less than 10 mg in a 2.5 mm rotor) made the acquisition of a good ^13^C spectrum quite problematic [50].

**Figure 8:**
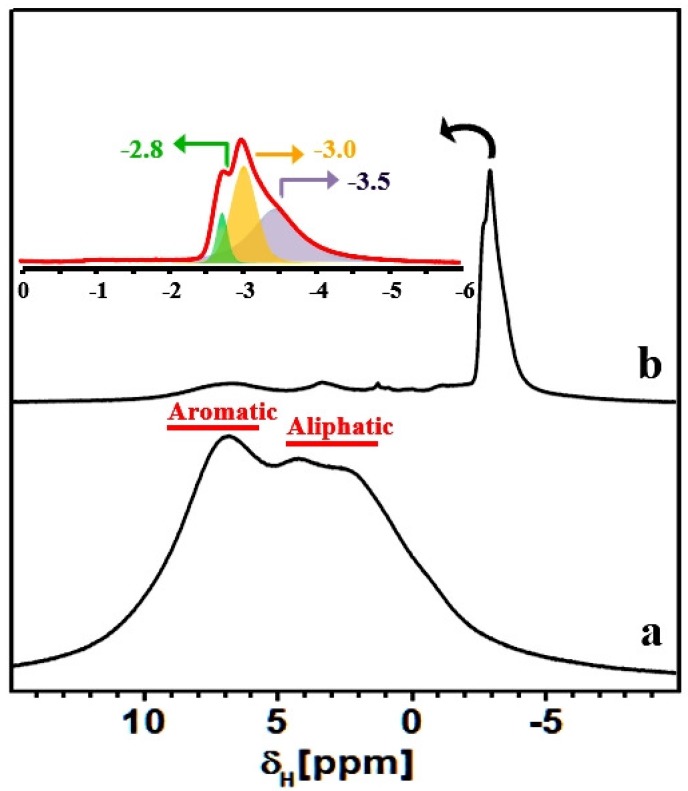
^1^H MAS NMR spectra of HCP-PS (a) and KPS-1 (b) recorded using a MAS rate of 15 kHz. Inset: zoomed KPS-1 spectrum.

Nevertheless, the ^13^C spectrum of KPS-1, obtained at a MAS rate of 30 kHz and shown in Figure 8, revealed a very broad resonance in the aromatic region and three narrow resonances in the aliphatic region. A typical resonance of a carbonized material appears at 129 ppm with a down-field shoulder at 150 ppm. The broad peak centered at 129 ppm is assigned to the presence of sp^2^ hybridized carbons sites as in graphitic material [51,52,53,54]. Furthermore, the broad shoulder centered at 150 ppm was due to the advancement of polycyclization of the benzene rings during carbonization. The narrow resonances at 7, 19, and 27 ppm were either due to the methyl and ethyl carbons generated upon the methylene disproportionation during carbonization, and/or the entrapped aliphatic carbon fragments in the hierarchical pores of the material.

To further test the above hypothesis, we employed the ^1^H MAS NMR technique, which proved particularly useful for studying molecular adsorption inside porous carbon materials. As shown in Figure 8, the HCP-PS polymeric material presents peaks due to both aliphatic and aromatic protons. Upon carbonization of the hyper-crosslinked polymeric material, intensity due to aromatic and aliphatic protons decreases dramatically, and intense multiple resonances appear at around −3 ppm (Figure 8 and the inset).

Such a strong up-field ^1^H shift (relative to TMS at 0 ppm) is highly unusual for aliphatic protons; however, proton species close to porous carbon surfaces can experience a reduced local magnetic field owing to the circulation of nearby delocalized π-electrons, leading to large chemically-shielded proton environments. The protonic species can be either structural or adsorbed in nature, and their chemical shifts can be influenced by the carbon ring currents, as has been reported for plane and curved graphene sheets, nanotubes, and fullerene-like elements [55,56,57]. As discussed above, these protonic species are probably associated with the aliphatic carbons fragments that appeared in the ^13^C NMR spectrum. However, a further NMR characterization is necessary for the detailing of chemical compositions of KPS-1, which is beyond the scope of this paper.

Then, ^13^C and ^1^H NMR spectroscopy unambiguously confirmed the hyper-crosslinking of the polystyrene units during the formation of HCP-PS. Moreover, carbon linkages such as benzylic units, derived from FDA between aromatic rings were identified. Finally, polycyclization of the benzene rings and the aliphatization of aromatic rings during carbonization were also determined.

### 3.4. Methane Adsorption

Before testing the methane adsorption in KPS-1, the synthesized fine powder needed to be compressed in tablets, allowing for the measure of the packing density (sometimes referred to as bulk density) used to estimate the volume-based gas uptake. After the compression, the KPS-1 packing density was 0.50 g/cm^3^, and the PSD and CPV were measured again on the compressed sample, without significant changes with respect to the as-synthesized powder up to pores of 200 Å width, showing that packing does not affect the micro- and mesopore network, while removing the interparticle voids and possibly part of the macropores.

**Figure 9:**
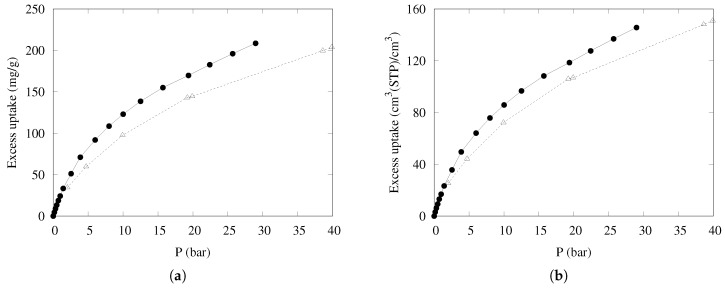
Excess adsorption isotherms at 298 K for CH_4_ on a (**a**) mass basis and a (**b**) volume basis in KPS-1 (solid circles) and LMA738 (open triangles) [18].

The uptake of methane on mass and volume bases, measured for compressed KPS-1 at 298 K with the procedure described in ref. [58], is shown in Figure 9, compared to the data reported in Ref. [18] for one of the best-performing porous carbons (LMA738), often used as a benchmark for methane adsorption. Note that both volume- and mass-based uptakes are of interest for large-scale applications, depending on whether the final weight or volume of the container is the limiting factor.

The results are very satisfying—at intermediate pressure (about 30 bar), both measures of uptake are markedly superior to the results of the best carbon-based materials described so far. The good mass-based uptake can be ascribed to the high microporosity (effective at low pressure), coupled to a large fraction of “small” mesopores (20–40 Å wide) but also to a long tail of wider mesopores, so that the adsorption is very high throughout the whole pressure range. Furthermore, the packing density is large enough to also maintain the good performance in the volume-based scale.

The adsorption performance of KPS-1 is compared to the results obtained at the same temperature for the best carbon-based materials in Table 2.

Another measure of the gas uptake has recently been suggested—namely, the storage capacity [18], designed to more accurately predict the performance of a given adsorber in real operative conditions. The storage capacity ($n_{stg}$), expressing the density of gas stored in an actual tank completely filled by the adsorber, is defined by the following equation:

(1) $$n_{stg}=n_{exc}+\rho_{gas}\left(1-\frac{\rho_{pack}}{\rho_{He}}\right)$$

Combining the excess uptake on a volume basis ($n_{exc}$) with the free gas density ($\rho_{gas}$), the quantity in parenthesis estimates the fraction of “dead volume” in the container from the adsorbent skeleton density ($\rho_{He}$) and its packing density ($\rho_{pack}$). KPS-1 skeleton density was measured by helium pycnometry as 2.32 g/cm^3^, which is a rather high value, confirming that the material is strongly amorphous, as suggested by Raman spectra.

**Figure 10:**
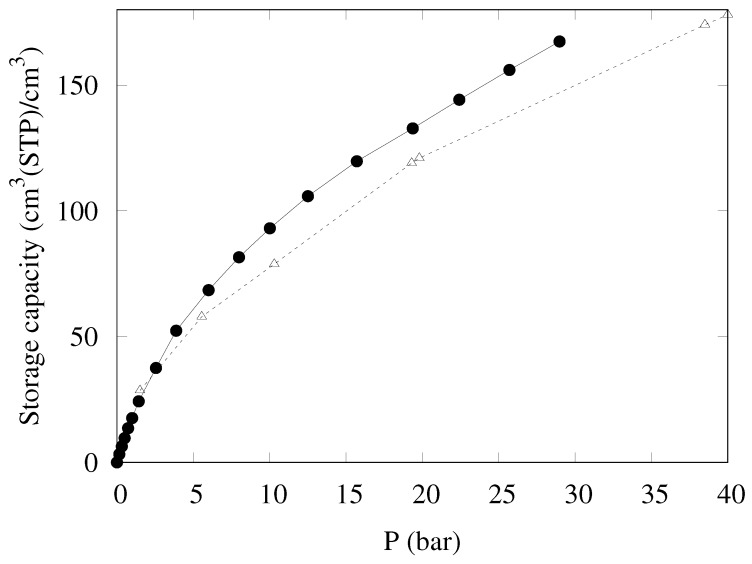
Storage density at 298 K for CH_4_ in KPS-1 (solid circles) and LMA738 (open triangles) [18].

The storage density provided by KPS-1 is reported in Figure 10; also in this case, KPS-1 performance is extremely good, as the storage capacity reaches 168 cm^3^(STP)/cm^3^ at 30 bar, larger than the best result published so far for a porous carbon (148 cm^3^(STP)/cm^3^ for LMA738 in Ref. [18]).

## 4. Conclusions

In this paper, we have described a new porous carbon material (KPS-1) able to adsorb a very large quantity of gaseous methane in a wide range of pressures. The material was obtained first by a Friedel-Crafts reaction on expanded polystyrene to get a highly hyper-crosslinked polymer (HCP-PS), and then with an activated carbonization of this polymer. Both HCP-PS and KPS-1 were carefully characterized by FTIR and Raman spectroscopy, which showed the degree of polymerization first and of carbonization after the activation process, as well as by SS-NMR, providing details on the nature of the chemical moieties formed in the process. Then, the porosity of both the polymer and the activated carbon were estimated with QSDFT analysis of nitrogen and CO_2_ adsorption isotherms.

Thanks to the optimal combination of micro- and mesoporosity and packing density, KPS-1 presented mass-based and volume-based methane uptakes, as well as storage capacities which were among the highest presented so far for this kind of material. KPS-1 was also synthesized starting from a commercial expanded polystyrene peanut through a cheap and easily scalable procedure, suggesting the possibility of a large-scale production from recycled materials. The KPS-1 adsorption capacity towards other gases, primarily CO_2_, as well as the test of various sources of expanded PS with different molecular weights of the starting material, will be described in future studies. 

## Figures and Tables

**Table 1:** Porous volume (cm^3^/g) of the produced materials.

Material	Total	Microporous	Mesoporous
HCP-PS	0.74	0.11	0.63
KPS-1	1.42	0.70	0.72
PC from ref. [34]	0.7	–	0.7
AC800 from ref. [35]	1.20	0.93	0.27

**Table 2:** Excess uptake of CH_4_ at 298 K in KPS-1 and in some of the best-performing materials.

Material	Pressure (Bar)	Mass-Based Uptake (g/g)	Volume-Based Uptake (cm^3^(STP)/cm^3^)	Bulk Density (g/cm^3^)	Ref.
KPS-1	30	0.209	146	0.50	this work
LMA738	35	0.191	142	0.53	[18]
PY100_700	35	0.178	150	0.60	[4]
DO100_700	35	0.182	160	0.62	[4]
K-PAF-1-750	35	0.207	n. a.	n. a.	[59]
KOH-corncob	35	0.25	n. a.	n. a.	[60]
Activ. monolith	30	0.136	154	0.80	[17,20]
